# Implementation of decarbonisation actions in general practice: a systematic review and narrative synthesis

**DOI:** 10.1136/bmjopen-2024-091404

**Published:** 2025-02-19

**Authors:** Ana Raquel Nunes, Florence Karaba, Olivia Geddes, Abi Bickerton, Helen Atherton, Frederik Dahlmann, Abi Eccles, Michael Gregg, Rachel Spencer, Helen Twohig, Jeremy Dale

**Affiliations:** 1 University of Warwick Warwick Medical School, Coventry, UK; 2 Primary Care Research Centre, University of Southampton, Southampton, UK; 3 University of Warwick Business School, Coventry, UK; 4 Nuffield Department of Primary Care Health Centre, Medical Sciences Division, Universiy of Oxford, Oxford, UK; 5 PPI co-applicant, NA, UK; 6 Keele University, Keele, UK

**Keywords:** Climate Change, Primary Healthcare, Primary Care, Health Services, HEALTH SERVICES ADMINISTRATION & MANAGEMENT

## Abstract

**Abstract:**

**Objectives:**

To summarise and synthesise existing literature on the implementation of decarbonisation actions in general practice, to outline the actions being implemented, factors influencing decarbonisation, identify evidence gaps and questions for future research.

**Design:**

A systematic review and narrative synthesis.

**Data sources:**

MEDLINE, Embase, CINAHL, Web of Science and ProQuest (grey literature) were searched for literature published up to 29 March 2024.

**Eligibility criteria for selecting studies:**

Studies of any design investigating the implementation of decarbonisation actions in general practice.

**Data extraction and synthesis:**

Two reviewers extracted data and conducted quality assessments using a mixed methods appraisal tool. Narrative synthesis was used to analyse findings.

**Results:**

Fifteen studies were included. Studies were primarily from the UK (n=5), followed by Australia (n=3), USA (n=2), Germany (n=2) and one each from France, Switzerland and Israel. Study designs were qualitative (n=7), quantitative (n=7) and one mixed methods. Participants included healthcare staff (n=7), patients (n=5), health stakeholders (n=2) and the general public (n=1). There was evidence of general practices adopting decarbonisation actions such as resource reuse, improved waste management, energy-efficient systems and preventive care to reduce overmedication, with strong leadership and institutional support being crucial for their success. However, barriers such as high costs, resource constraints and limited awareness among clinicians and patients highlighted the need for enhanced communication, education and the structured promotion of initiatives to improve patient and community engagement.

**Conclusions:**

There is limited evidence on the implementation of decarbonisation actions in general practice. A range of factors may impact on the extent to which implementation occurs. Addressing these will be crucial for effectively promoting and scaling decarbonisation actions in general practice. Future research should focus on understanding the role of institutional context, evaluating the real-world impact of interventions on greenhouse gas emissions and exploring patient and community involvement.

**PROSPERO registration number:**

CRD42023470889.

**STRENGTHS AND LIMITATIONS OF THIS STUDY**

- This study uses a systematic review methodology to examine the integration of decarbonisation actions into general practice.
- It provides a comprehensive and up-to-date analysis of the implementation of decarbonisation actions in general practice, drawing from a diverse range of international literature.
- The focus on studies from 2007 onwards aligns with significant developments in global climate change policy.
- The restriction to studies published in English may introduce language bias and limit the generalisability of the findings.

## Background

 ‘Tackling climate change could be the greatest global health opportunity of the 21st century.’^1^ Through comprehensive mitigation efforts aimed at reducing greenhouse gas (GHG) emissions, coupled with robust adaptation strategies to address the unavoidable impacts of climate change, there is the potential to transform healthcare systems and improve health outcomes worldwide.^2^,^7^ Mitigation efforts, such as promoting decarbonisation actions, reducing carbon emissions and adopting low carbon technologies, may also improve air quality, reduce the burden of chronic diseases and enhance overall well-being.^8^ Furthermore, adaptation measures, including strengthening healthcare infrastructure, enhancing disaster preparedness and implementing resilience-building initiatives, can help healthcare systems better cope with the changing climate and mitigate the health risks associated with extreme weather events, infectious diseases, climate anxiety and other climate-related challenges.^9^,^11^ By embracing both mitigation and adaptation strategies within the healthcare sector, there is an opportunity to protect health, build resilient communities and create a sustainable future.^12 13^

Primary care, as the initial point of contact in healthcare, has a pivotal role in tackling these challenges.^14^ In the UK, the healthcare sector is responsible for around 4–5% of the total GHG emissions, with primary care being responsible for around 23% through direct care delivery, staff and patient travel and other related services.^15^,^18^ Consequently, addressing primary care’s environmental impact is crucial for overall healthcare sustainability, as highlighted by the 2020 National Health Service (NHS) report on delivering net zero.^17^ Achieving net zero requires leadership and systemic behaviour change at all levels of healthcare.^1 3 6 19^ However, the British Medical Association has observed that primary care lacks detailed guidance on its role in achieving net zero carbon emissions within healthcare.^20^ Furthermore, the distributed and varied organisational structure of primary care presents unique challenges to implementing sustainability initiatives.^21^

Targeted interventions and the adoption of decarbonisation actions in primary care offer the potential to reduce the sector’s carbon footprint, improve patient outcomes, foster community resilience and inspire other healthcare sectors to follow suit.^5 22 23^ In the UK, as the foundation of primary care and gateway to other healthcare services in the NHS, the role of general practice is therefore significant.^24 25^

Despite these opportunities, scoping searches identified no prior systematic reviews examining the implementation of decarbonisation actions in general practice or family practice. The aim of this study is to address this gap in knowledge by systematically exploring the existing body of empirical research on the implementation of decarbonisation actions in general practice. Specifically, this review summarises and synthesises existing literature, identifies factors influencing decarbonisation (eg, patient and community engagement), highlights evidence gaps and outlines questions for future research. By examining how and why decarbonisation actions are implemented, this review seeks to inform the commissioning and delivery of general practice and family practice services, ultimately facilitating the transition toward sustainable healthcare.

## Method

This systematic review was conducted following a predefined protocol.^26^ It uses a mixed-methods design and is reported according to the Preferred Reporting Items for Systematic Reviews and Meta-Analysis (PRISMA) framework.^27^

### Patient and public involvement

Patient and public representatives were integral to the review. They were involved in the design, development and conduct of this review. A patient and public representative provided feedback on drafts and is a coauthor.

### Eligibility criteria

The eligibility criteria were structured according to the PICO framework. Population: studies investigating decarbonisation actions in general practice (or equivalent in non-UK settings). Intervention: any decarbonisation actions aimed at reducing carbon emissions within primary care. Comparator: current decarbonisation actions. Outcome: the extent and effectiveness of decarbonisation actions and factors influencing their implementation.

Eligible decarbonisation actions were defined as initiatives aimed at reducing carbon emissions within general practice settings. Bottom-up (micro-level) and top-down (meso-level and macro-level) dimensions were considered eligible.

The inclusion criteria for the review were any study design; studies that investigated the implementation of decarbonisation actions in general practice (or equivalent in non-UK studies); studies published in English from 2007 onwards. Studies were excluded if they were published as a poster, letter, conference abstract and if based in community pharmacy, walk-in centres, dental and optometry (eye health) services (or equivalent in non-UK studies). In this review, we define primary care as comprising general practice, community pharmacies, dental services and eyecare through optometry, with general practice described as a primary care medical service delivered by general practitioners (GPs) and the multidisciplinary teams who are based within general practice.

### Search strategy

Databases were searched from January 2007 to March 2024 and included MEDLINE, Embase, Web of Science and CINAHL. Searches for grey literature were also conducted in ProQuest. The selected date coincides with the UN climate change conference where negotiations on a successor to the Kyoto Protocol began. Search strategies can be found in online supplemental table 1. Forwards and backwards citation searches were undertaken on all included articles. Non-English studies were identified and screened using translation software to determine eligibility.

### Study selection and data extraction

After duplicates were removed, two reviewers screened studies independently at title and abstract stage and at full text stage using Rayyan (systematic review management software).^28^

A data extraction form was developed where key elements of studies were captured independently by the two reviewers. Data extraction included study characteristics, intervention details, outcomes and implementation factors. Discrepancies were resolved through discussion with a third reviewer. Double data extraction was performed to ensure accuracy.

### Outcomes

The primary outcomes of interest were the types of decarbonisation actions implemented, including telehealth, deprescribing, respiratory inhalers and single-use disposables. Secondary outcomes included factors influencing the adoption, implementation and integration of decarbonisation actions at institutional, organisational, professional and patient spheres.

### Quality assessment

The Mixed Methods Appraisal Tool (MMAT), designed for reviews where study designs are mixed and individual studies use mixed methods, was used to assess the quality of included studies.^29^ Two reviewers independently assessed the quality of the studies, and discrepancies were addressed through discussion. Studies were categorised as high, medium or low quality, depending on how many MMAT criteria were met. An overall quality rating was determined for contextual information only, and studies were not excluded on this basis.

### Data synthesis

A narrative synthesis approach was used due to the diversity of designs of included studies, allowing for systematic analysis of studies with different designs by considering their similarities and differences.^30^ An iterative approach was applied, initially describing the characteristics and key findings of included studies, which were then organised to identify patterns. Patterns were explored within and between studies.

## Results

The search strategy identified 188 peer-reviewed and grey literature studies. After duplicates were removed, there were 168 studies to screen at title and abstract level; 48 studies were included for full-text screening, out of which 15 studies were included in this review.^31^,^45^ There were no eligible articles identified from the grey literature database search. The screening process, numbers and reason for exclusions can be found in the PRISMA flowchart^27^ (online supplemental figure 1). The main characteristics of included studies can be found in table 1.

**Table 1:** Characteristics of included studies (Note: the setting of the studies is general practice or its equivalent in non-UK studies)

First author, year	Country	Setting and participants	Study design
Legrand, 2023^31^	France	12 general practices, n=12 GPs	Qualitative design using face-to-face or phone semi-structured interviews.
Pavli, 2023^32^	Australia	3 general practices, n=23 staff (nurses, administrative staff and doctors)	Qualitative design, case study using semi-structured interviews and observations relating to environmental sustainability.
Muller, 2023^33^	USA	Various primary care practices/clinics, n=103 primary care clinicians (including resident and attending physicians, clinical psychologists, nurse practitioners and physicians’ assistants)	Quantitative design using cross-sectional questionnaire survey.
Andrews, 2013^34^	UK	1 general practice, n=306 patients (survey); n=12 NHS clinical staff (focus group 1); n=13 NHS non-clinical staff (focus group 2)	Mixed methods design, case study, using survey and two focus groups. The focus groups followed a semi-structured topic guide. Carbon footprint was estimated using the ArcInfo GIS software package.
Griesel, 2023^35^	Germany	6 primary care practices, n=27 patients	Qualitative design using semi-structured interviews and cross-sectional survey.
Fehrer, 2023^36^	Germany	Various primary care practices, n=40 physicians, medical assistants, health scientists and experts on the healthcare system	Qualitative exploratory design using semi-structured guide-based interviews and focus groups.
Foley, 2023^37^	Australia	Nature-based prescribers and providers, n=13 health stakeholders (health service providers and managers)	Qualitative descriptive design using semi-structured interviews.
Andre, 2022^38^	Switzerland	Various general practices, n=497 GPs	Quantitative design using cross-sectional survey.
Boland and Temte, 2019^39^	USA	4 family medicine and community health clinics, n=403 patients; n=58 family physicians	Quantitative design using cross-sectional survey.
Maughan, 2016^40^	UK	Social prescribing intervention ‘The Connect project’, n=30 Connect project group; n=29 (control group)	Quantitative design using retrospective observational data.
Woodcock, 2021^41^	UK	Salford Lung Study in Asthma, n=2236 subset of study participants	Quantitative design using carbon footprint analysis and clinical outcomes analysis.
Wild, 2023^42^	Australia	3 Australian Regional Training Organisations, n=879 GP registrars	Quantitative design using cross-sectional questionnaire.
Robinson, 2020^43^	UK	Social prescribing intervention ‘The Connect project’, n=114 GPs; n=170 nature-based organisation participants	Quantitative design using online cross-sectional questionnaire.
Guggenheim, 2016^44^	Israel	1 general practice, n=107 patients	Quantitative using questionnaire.
Sun, 2023^45^	UK	1 region of the UK, n=34 stakeholders, n=64 members of the public	Qualitatively design using observations and shadowing, workshops and semi-structured interviews.

GPsgeneral practitionersNHSNational Health Service

### Characteristics of included studies

Studies were from the UK (n=5),^34 40 41 43 44^ Australia (n=3),^32 36 42^ USA (n=2),^33 39^ Germany (n=2),^35 36^ France (n=1),^29^ Switzerland (n=1)^38^ and Israel (n=1).^44^ Most were either of qualitative (n=7)^3132 35^,^37 44 45^ or quantitative design (n=7),^3338^,^43^ with one mixed methods study included (n=1).^34^ Cross-sectional surveys (n=7)^3335 38 39 42^,^44^ and semi-structured qualitative interviews (n=6)^3132 35^,^37 45^ were the most prominent methods used. Fewer studies used focus groups (n=3),^34 36 45^ observations (n=2),^32 45^ retrospective observational study (n=1)^40^ and carbon footprint analysis and clinical outcomes analysis (n=1).^41^ Studies collected data from a range of participants, including staff (n=7): GPs (n=3),^31 38 43^ other healthcare staff (n=3)^32 36 43^ and GP registrars (n=1)^42^; patients (n=5),^34 35 39 41 44^ and health stakeholders (n=2),^37 45^ the general public and stakeholders (n=1).^45^

### Quality assessment

According to the MMAT guide,^29^ 10 studies were rated high quality (green),^3132 34^,^37 40^ 4 were rated as moderate quality (orange)^33 38 39 43^ and 1 was rated low quality (red).^44^ Quality assessment ratings for each study can be found in online supplemental table 2.

### Type of decarbonisation actions

In all included studies,^31^,^45^ there is evidence of general practice integrating decarbonisation actions into their operations to reduce carbon emissions and promote environmental sustainability. Decarbonisation actions identified varied across settings and methodologies. Some studies derived these actions from qualitative interviews,^3132 35^,^37 45^ focus groups^34 36^ or observational studies.^32 45^ Actions included reorganising practice operations to promote reuse of resources,^31 36^ improving waste management through selective sorting^31 32 36^ and revising medical prescriptions to prevent overmedication and focus on preventive care.^31 33 40 41^ These measures aimed to reduce healthcare costs and environmental pollution. However, implementation details and evaluations of effectiveness were often missing.

Energy-efficient systems, such as LED lighting and upgraded heating, were commonly adopted, particularly in countries with supportive policies.^31 32 36 40^ Strategies to minimise patient travel emissions included promoting telemedicine, public transport, walking, carpooling, complemented by administrative adjustments to optimise appointment scheduling and prescription collection.^34^ Despite this, the level of patient uptake and evaluation of these strategies was unclear. In Germany, climate-sensitive health counselling provided patients with education about climate change and health and encouraged eco-friendly behaviours.^36^ In Australian practices, the integration of nature prescriptions was used to encourage outdoor activities to improve patient health while reducing environmental impact, highlighting the importance of community collaboration and robust clinical processes in achieving sustainable healthcare outcomes.^37^

One study^40^ demonstrated that social prescribing can reduce healthcare use, including secondary-care referrals, thereby lowering the carbon footprint. Another study^41^ found that switching patients with asthma from pressurised metered-dose inhalers to dry powder inhalers significantly reduced the carbon footprint without compromising asthma control, suggesting that environmentally friendly options can be effectively incorporated into patient care.

### Institutional and policy support

Institutional and policy support emerged as crucial enablers for decarbonisation efforts in general practice. Financial incentives at both individual and practice levels facilitated actions such as upgrading facilities and adopting sustainable practices.^36 37^ However, specific examples of these incentives were often vague. Supportive policies were also essential for the adoption of decarbonisation actions, with barriers such as the lack of clear guidance in some regions hindering widespread implementation.^36 37^ In some cases, regional policies and frameworks, such as the World Organization of Family Doctors (WONCA) declaration, provided guidance and motivated GPs to integrate climate change considerations into their practices.^36 37^ Nonetheless, the lack of clear and region-specific directives hindered broader implementation. Effective decarbonisation also required system-level changes, including better networking and centralisation of sustainability efforts.^32 36^ For a summary, see table 2.

**Table 2:** Factors influencing the adoption, implementation and integration of decarbonisation actions

Factors	Description
**1. Institutional and policy support**	
1.1. Financial incentives and policies	Financial incentives are essential for the adoption of decarbonisation actions, but inconsistent policy guidance in some regions acts as a barrier.^36 37^
1.2. Frameworks and declarations	Guidelines such as the WONCA declaration motivate GPs to integrate climate change considerations into their practices by providing structured guidelines and strategic vision.^36 37^
1.3. System level changes	Effective decarbonisation requires better networking and centralisation of sustainability efforts to ensure coherence and efficiency across the healthcare system.^32 36^
**2. Organisational leadership, support and constraints**	
2.1. Leadership and culture	Proactive leadership and a culture that values sustainability are critical for driving successful decarbonisation efforts within general practices.^32 42^
2.2. Practice management	Effective leadership and staff engagement are essential for integrating decarbonisation actions into daily practice activities.^32 42^
2.3. Resource constraints	High costs and resource limitations hinder the adoption of sustainable measures, requiring financial support and cost-effective solutions.^32 36^
**3. Professional knowledge, awareness and engagement**	
3.1. Knowledge and awareness	Clinician awareness of climate change impacts is crucial, but many lack specific knowledge and feel uncomfortable discussing it with patients.^33 38 39^
3.2. Education and training	Enhancing clinician competence through targeted education and training on decarbonisation is needed.^33 44^
3.3. Personal environmental consciousness	GPs who are environmentally conscious personally are more likely to adopt decarbonisation actions professionally.^33 36^
3.4. Variation in awareness and engagement	Significant differences exist among clinicians, with high willingness to learn but low comfort in counselling patients on climate-related issues.^38 39^
3.5. Preferences and acceptance	Variability in acceptance of sustainability roles and measures, with constraints including limited awareness, funding and patient motivation.^42 43^
**4. Patient and community engagement**	
4.1. Patient discussions and barriers	Many GPs discuss climate change with patients, but barriers such as time constraints and lack of recommendations limit these discussions.^35 38 39^
4.2. Patient perception and information sources	Patients believe climate change affects health but rely on non-medical sources for information.^38 39^
4.3. Community engagement in activities	Local communities engage in nature-based activities, but awareness of initiatives like Green Social Prescribing is limited.^43 45^
4.4. Information gap	Patients trust physicians but do not view them as primary sources of environmental information, relying instead on news outlets, social media and personal networks.^38 39^

GPsgeneral practitionersWONCAWorld Organization of Family Doctors

### Organisational leadership, support and constraints

Leadership support for environmental sustainability proved pivotal, as demonstrated in Australian practices where management buy-in significantly influences the success of these initiatives.^32^ Strong leadership and a supportive workplace culture that values sustainability were critical for successful decarbonisation.^32 42^ Practices with proactive leadership and a culture prioritising environmental responsibility tended to achieve higher engagement and successful implementation of green practices. Effective practice management, including supportive leadership and staff engagement, was essential for integrating decarbonisation actions into general practice activities.^32 36 42^ However, high costs and resource constraints limited the ability of practices to adopt sustainable measures; financial support and cost-effective solutions were needed to overcome these barriers.^32 43^ Such barriers were frequently cited but seldom quantified, with few studies providing detailed evaluations of these costs or proposing cost-effective alternatives.

One study^38^ found that Swiss GPs believed they can serve as role models for sustainability and advocate for stronger outreach from medical associations on climate change and health. For a summary, see table 2.

### Professional knowledge, awareness and engagement

Knowledge and awareness of climate change and its health impacts among general practice clinicians were identified as crucial for promoting decarbonisation actions.^33 36 42^ However, there was evidence that while clinicians acknowledged the existence and threat of climate change, they may lack specific knowledge and felt uncomfortable discussing it with patients due to insufficient training and a lack of practical tools.^39^ Their knowledge on specific topics such as planetary health was limited.^38^ In one study, despite high willingness to learn more, only 17% of US physicians felt comfortable counselling patients on climate-related health issues.^39^ Enhancing clinician competence through education and training on decarbonisation was found to be essential, with educational interventions aimed at enhancing knowledge and skills being recommended but not extensively evaluated.^33 36 42^ Moreover, GPs’ personal environmental consciousness appeared to influence their professional practices, with those engaged in sustainable activities at home more likely to adopt green measures in their practices.^33 36 42^ GPs perceived themselves as influential in promoting sustainability to both patients and colleagues.^33 42^

Preferences and acceptance of such measures among general practice professionals appeared to vary. One study^42^ reported that most GP registrars support leadership roles in environmental sustainability within their practices. Another^43^ emphasised that effective green prescribing depends on the availability of services and green spaces, with GPs in less deprived areas more likely to prescribe nature-based interventions. However, significant constraints existed, including limited awareness, funding and patient motivation, which can hinder the widespread adoption of green prescribing.^43^ For a summary, see table 2.

### Patient and community engagement

Patient and community engagement may play a pivotal role in promoting decarbonisation actions within general practice, but it was underexplored in most studies. One study^38^ reported that 78% of GPs in Switzerland discussed climate change with patients, with 44% doing so in over 10% of their consultations. However, many GPs felt uncomfortable advising on this topic due to barriers such as time constraints and lack of clear clinical recommendations, which limited these interactions. While some GPs actively discussed climate-related health issues, the frequency and effectiveness of these discussions varied widely. Another study^39^ reported that 44% of patients in the USA believed climate change affects their community’s health, but only 6% considered their physician a top source of environmental information, indicating underutilisation of physicians as sources of information despite high patient trust.

One study^45^ revealed that while local communities engaged in nature-based activities, awareness of green social prescribing (GSP) was limited, with most participants learning about activities through informal channels such as social media rather than formal referrals.

Patients, while concerned about environmental issues, often relied on non-medical sources for environmental information, highlighting a missed opportunity for GPs to act as trusted advisors on climate-related health issues. Two studies^38 39^ indicated that patients' primary sources of environmental information include news outlets, social media and family and friends, highlighting a gap between patient concern and the information provided by general practice professionals. Engagement strategies, such as nature-based activities and green social prescribing, showed promise but faced challenges related to patient awareness and accessibility. For a summary, see table 2.

### Implementation in practice

Implementation strategies varied and were inconsistently reported. Some practices were reported as achieving success through strong leadership and organisational buy-in, fostering a culture prioritising sustainability.^32 42^ Others struggled with limited staff and patient engagement^32 34 37 45^ or unclear guidance.^36 37 43^ For example, patient travel reduction initiatives often lacked monitoring systems to evaluate their effectiveness.^34^ Similarly, green prescribing depended heavily on the availability of local resources, which varied significantly across settings.^43^

## Discussion

### Summary

This systematic review identified 15 studies of variable quality and scale undertaken in seven different countries, with most having been published since 2022. Its findings indicate ways through which general practices are adopting decarbonisation actions to reduce carbon emissions and promote environmental sustainability. This includes addressing resource reuse,^31 32^ improved waste management,^31 32 36^ energy-efficient systems^31 32^ and preventive care to reduce overmedication.^31 41^ There was also evidence of strategies to minimise patient travel emissions, such as telemedicine,^34^ educate patients on climate change through climate-sensitive health counselling^34^,^36^ and integrate nature prescriptions into everyday healthcare practices.^37^

However, the review also identified significant barriers to implementation, such as high costs, resource constraints and limited awareness among both clinicians and patients.^32 36 38 39^ Institutional support, including financial incentives and clear policies, can overcome barriers to implementation.^36 37^ Strong leadership and a supportive organisational culture foster the adoption of decarbonisation actions.^32 42^ Education and training for clinicians on environmental sustainability can also help equip them to promote decarbonisation actions and engage with patients effectively.^33 34 42 44^ Patient and community engagement are also crucial, particularly through structured promotion.^42 44^ Patients often rely on non-medical sources for environmental information, highlighting an opportunity for improved communication within general practice settings.^36 39^ Patient-centred communication that links climate change to health and structured promotion of green prescribing can improve patient and community engagement in decarbonisation actions.^35 39 45^

### Strengths and limitations

This review addresses a critical gap in understanding the integration of decarbonisation actions in general practice and is the first systematic review to tackle this topic. Additionally, it provides a comprehensive and up-to-date analysis of the implementation of decarbonisation actions in general practice, drawing insights from internationally diverse sources and perspectives. Despite a comprehensive search and an iterative process to widen the scope, a relatively small number of papers were identified (n=15). While searches were restricted from 2007 onwards, only three of the included studies were published pre-2020. The inclusion of studies published only in English is a limitation, which may limit the generalisability of findings and may have excluded valuable evidence from studies published in other languages.

### Comparison with other literature

The findings align with existing literature on decarbonisation actions in healthcare, emphasising the feasibility and benefits of decarbonisation actions such as resource optimisation,^31 32^ improved waste management,^31 36^ adopting energy-efficient systems and promoting preventive care^31 41^ to reduce carbon emissions and healthcare costs. Similarly, some research^46^ has highlighted the positive impact of streamlined systems and incentives, but noted challenges such as political affiliation and organisational constraints, which are echoed in this review through the need for leadership support and financial considerations.^32^

The emphasis on reducing patient travel emissions through telemedicine and optimising appointment scheduling^34^ resonates with those who advocate for telemedicine to mitigate environmental impacts.^47^ Additionally, the implementation of climate-sensitive health counselling and nature prescriptions in general practice^36 37^ parallels findings from others^48^ on the effectiveness of nature-based interventions in community health.

Institutional and policy support are crucial, with guidelines such as the WONCA declaration^36 37^ providing essential guidance, mirroring the need for systemic changes and better networking noted in the literature.^32 34^ The pivotal role of leadership and a supportive workplace culture^32 36^ is consistent with others,^49^ emphasising universal leadership significance across general practices.

Professional engagement through enhanced education and training on environmental sustainability^33 38 44^ is essential in addressing the gap between climate change awareness and clinician behaviour.^50^ Despite high awareness, the discomfort in discussing climate-related health issues^39^ indicates a systemic issue requiring targeted education and cultural change.^42^

Patient and community engagement are vital, with findings indicating that structured promotion of GSP^45^ and improved communication strategies^35 39^ are necessary to bridge the gap between patient concern and the information provided by general practice professionals. These insights align with the broader literature, underscoring the need for tailored approaches to sustainability in healthcare.^3 5 51^ Overall, the comparison reveals consistent themes across general practice, hospital and community care settings, highlighting the universal challenges and facilitators of decarbonisation actions.

### Implications for decarbonisation and future research

General practice demonstrates the potential to integrate decarbonisation actions effectively, reducing carbon emissions and promoting environmental sustainability. However, there is a need for financial support and cost-effective solutions to overcome the high costs and resource constraints that often limit the adoption of sustainable measures.^32 36 37^ Practical measures, such as resource reuse, improved waste management^31 36^ and energy conservation through the adoption of energy-efficient systems such as LED lighting,^31 32^ not only contribute to environmental goals but also offer financial benefits by reducing healthcare costs associated with overmedication and inefficient energy use.

Institutional and policy support are critical for scaling up decarbonisation efforts. Financial incentives and clear guidelines, such as those provided by the WONCA declaration, are essential to motivate and guide GPs in integrating climate change considerations into their practices.^36 37^ Future research should explore strategies to foster strong leadership and supportive workplace cultures that prioritise environmental responsibility, including evaluating the effectiveness of these policies and identifying best practices for systemic changes, including better networking and centralisation of sustainability efforts.^32 36 37^

Professional engagement through education and training is also crucial.^33 36 42^ While many clinicians acknowledge the threat of climate change, they often lack specific knowledge and feel uncomfortable discussing it with patients.^39^ Enhancing clinician competence through targeted education on environmental sustainability can bridge this gap. Moreover, personal factors, such as parenthood, can motivate clinicians to adopt and advocate for decarbonisation actions, suggesting that personal triggers could be leveraged in professional training programmes.^33 36 38 42^

Patient and community engagement is essential for the success of decarbonisation actions. A patient-centred approach that underscores health co-benefits of climate-friendly lifestyles as well as the integration of initiatives such as GSP within community health can enhance engagement and acceptance.^35 43 45^ Future research should investigate the most effective communication and education strategies to bridge this gap and enhance the use of general practice professionals as trusted sources of environmental information.

The findings from this review have significant implications for health policy, clinical practice and patient care, aligning well with behaviour change frameworks such as the Theoretical Domains Framework (TDF)^51^ and Normalisation Process Theory (NPT).^52 53^ Given that decarbonisation actions in general practice are influenced by institutional, organisational and individual behavioural factors, as well as contextual factors like patient views and experiences, both TDF^54^ and NPT^52 53^ can be used to structure future data collection and analysis. Such a combined approach will systematically identify cognitive, affective and environmental determinants relevant to implementing decarbonising actions within general practice and understand the dynamic social processes involved.^52^,^54^

Additionally, while this review identifies ways in which general practices have made strides in integrating decarbonisation actions, the extent to which widespread implementation is occurring remains limited.^32 36^ Future research should focus on implementation strategies, including strengthening leadership, providing financial and policy support, enhancing professional education and improving patient and community engagement. Tailored approaches that consider the unique contexts of different general practice settings and patient populations will be crucial for the widespread adoption, scaling up and success of decarbonisation efforts.^32 36 43^

Finally, future research could explore the role of removing low-value care, such as inappropriate testing and prescribing, as a crucial strategy for decarbonisation. Tackling unnecessary healthcare practices not only contributes to emission reductions but also provides significant co-benefits, including improved patient safety and reduced healthcare costs.

## supplementary material

10.1136/bmjopen-2024-091404 online supplemental figure 1

10.1136/bmjopen-2024-091404 online supplemental table 1

10.1136/bmjopen-2024-091404 online supplemental table 2

## Data Availability

Data are available upon reasonable request.
