# Supplementary material for: Implementation of decarbonisation actions in general practice: a systematic review and narrative synthesis
Source: BMJ Open. 2025 Feb 19;15(2):e091404. doi: 10.1136/bmjopen-2024-091404 (PMC11840891; doi:10.1136/bmjopen-2024-091404)
Supplement: online supplemental figure 1 [file bmjopen-15-2-s001.docx]

**Supplementary Figure 1**

**PRISMA flowchart**

PRISMA diagram showing screening and included studies

Duplicate records removed

(n =20)

Records identified from databases

(n = 188)

**Identification**

Records excluded

(n =120)

Records screened

(title and abstract)

(n =168)

**Screening**

Full-text articles assessed for eligibility

(n =48)

Full-text articles excluded

(n =38)

Records identified from reference searches

(n = 5)

Studies included in review

(n = 15)

**Included**

*From:*  Page MJ, McKenzie JE, Bossuyt PM, Boutron I, Hoffmann TC, Mulrow CD, et al. The PRISMA 2020 statement: an updated guideline for reporting systematic reviews. BMJ 2021;372:n71. doi: 10.1136/bmj.n71
