# Supplementary material for: Implementation of decarbonisation actions in general practice: a systematic review and narrative synthesis
Source: BMJ Open. 2025 Feb 19;15(2):e091404. doi: 10.1136/bmjopen-2024-091404 (PMC11840891; doi:10.1136/bmjopen-2024-091404)
Supplement: online supplemental table 1 [file bmjopen-15-2-s002.docx]

**Supplementary Table 1**

**Search strategies**

| **Concept** | **Description** | **Search Terms** |
| --- | --- | --- |
|  | Setting | “General practice” OR “primary care” OR “family practice” OR “family clinic” OR “family medicine” OR “community health” OR “medical centre” OR “Primary healthcare” OR “Primary health care” |
|  | Implementation | “Greenhouse gas*” OR “GHG” OR “Net zero” OR “Net-zero” OR “climate change” OR “carbon emissions” OR “*carbon footprint” OR “environmental sustainability” |
|  | Intervention | “implement*” OR “strateg*” OR “action*” OR “intervention*” OR “policies” OR “policy” OR “solution*” OR “plan” |
|  | Subjects | “staff” OR “patient*” OR “team*” OR “employee*” |

**Search term examples taken from two databases with key mesh terms:**

|  | **MEDLINE** |
| --- | --- |
| 1 | ("General practice" or "primary care" or "family practice" or "family clinic" or "family medicine" or "community health" or "medical centre" or "Primary healthcare" or "Primary health care").mp. |
| 2 | limit 1 to (english language and yr="2007 - 2023") |
| 3 | ("Greenhouse gas*" or "GHG" or "Net zero" or "Net-zero" or "climate change" or "carbon emissions" or "*carbon footprint" or "environmental sustainability").mp. |
| 4 | limit 3 to (english language and yr="2007 - 2023") |
| 5 | 2 and 4 |
| 6 | ("implement*" or "strateg*" or "action*" or "intervention*" or "policies" or "policy" or "solution*" or "plan").mp. |
| 7 | limit 6 to (english language and yr="2007 - 2023") |
| 8 | ("staff" or "patient*" or "team*" or "employee*").mp. |
| 9 | limit 8 to (english language and yr="2007 - 2023") |
| 10 | 7 and 9 |
| 11 | 5 and 10 |

**Web of Science**

(“General practice” OR “primary care” OR “family practice” OR “family clinic” OR “family medicine” OR “community health” OR “medical centre” OR “Primary healthcare” OR “Primary health care” ) (Topic) and (“Greenhouse gas*” OR “GHG” OR “Net zero” OR “Net-zero” OR “climate change” OR “carbon emissions” OR “*carbon footprint” OR “environmental sustainability”) (Topic) and (“implement*” OR “strateg*” OR “action*” OR “intervention*” OR “policies” OR “policy” OR “solution*” OR “plan") (Topic) and (“staff” OR “patient*” OR “team*” OR “employee*”) (Topic)

**ProQuest**

noft("General practice" OR "primary care" OR "family practice" OR "family clinic" OR "family medicine" OR "community health" OR "medical centre" OR "Primary healthcare" OR "Primary health care") AND noft("Greenhouse gas*" OR "GHG" OR "Net zero" OR "Net-zero" OR "climate change" OR "carbon emissions" OR "carbon footprint" OR "environmental sustainability") AND noft("implement*" OR "strateg*" OR "action*" OR "intervention*" OR "policies" OR "policy" OR "solution*" OR "plan") AND noft("staff" OR "patient*" OR "team*" OR "employee*")

Source type: Blogs, Podcasts, & Websites, Books, Conference Papers & Proceedings, Dissertations & Theses, Magazines, Reports

Language: English

**CINAHL**( “General practice” OR “primary care” OR “family practice” OR “family clinic” OR “family medicine” OR “community health” OR “medical centre” OR “Primary healthcare” OR “Primary health care” ) AND ( “Greenhouse gas*” OR “GHG” OR “Net zero” OR “Net-zero” OR “climate change” OR “carbon emissions” OR “*carbon footprint” OR “environmental sustainability” ) AND ( “implement*” OR “strateg*” OR “action*” OR “intervention*” OR “policies” OR “policy” OR “solution*” OR “plan” ) AND ( “staff” OR “patient*” OR “team*” OR “employee*” )

Limiters - Publication Date: 20070101-20231231; English Language; Language: English
Expanders - Apply equivalent subjects
Search modes - Find all my search terms
