# Supplementary material for: Implementation of decarbonisation actions in general practice: a systematic review and narrative synthesis
Source: BMJ Open. 2025 Feb 19;15(2):e091404. doi: 10.1136/bmjopen-2024-091404 (PMC11840891; doi:10.1136/bmjopen-2024-091404)
Supplement: online supplemental table 2 [file bmjopen-15-2-s003.docx]

**Supplementary Table 2**

**MMAT Quality assessment tables for included papers**

|  |  | **Legrand et al 2023** | **Pavli et al 2023** | **Muller et al 2023** | **Andrews et al 2013** | **Griesel et al 2023** | **Fehrer et al 2023** | **Foley et al 2023** | **Andre et al 2022** | **Boland and Temte 2019** | **Maughan et al 2016** | **Woodcock et al 2021** | **Wild et al 2023** | **Robinson et al 2020** | **Guggenheim 2016** | **Sun et al 2023** |
| --- | --- | --- | --- | --- | --- | --- | --- | --- | --- | --- | --- | --- | --- | --- | --- | --- |
| **SCREENING QUESTIONS** | S1. Are there clear research questions? | Yes | Yes | Yes | Yes | Yes | Yes | Yes | Yes | Yes | Yes | Yes | Yes | Yes | Yes | Yes |
|  | S2. Do the collected data allow to address the research questions? | Yes | Yes | Yes | Yes | Yes | Yes | Yes | Yes | Yes | Yes | Yes | Yes | Yes | Yes | Yes |
| **1. QUALITATIVE STUDIES** | 1.1. Is the qualitative approach appropriate to answer the research question? | Yes | Yes | N/A | Yes | Yes | Yes | Yes | N/A | N/A | N/A | N/A | N/A | N/A | Yes | Yes |
|  | 1.2. Are the qualitative data collection methods adequate to address the research question? | Yes | Yes | N/A | Yes | Yes | Yes | Yes | N/A | N/A | N/A | N/A | N/A | N/A | Yes | Yes |
|  | 1.3. Are the findings adequately derived from the data? | Yes | Yes | N/A | Yes | Yes | Yes | Yes | N/A | N/A | N/A | N/A | N/A | N/A | Can't tell | Yes |
|  | 1.4. Is the interpretation of results sufficiently substantiated by data? | Yes | Yes | N/A | Yes | Yes | Yes | Yes | N/A | N/A | N/A | N/A | N/A | N/A | Can't tell | Yes |
|  | 1.5. Is there coherence between qualitative data sources, collection, analysis and interpretation? | Yes | Yes | N/A | Yes | Yes | Yes | Yes | N/A | N/A | N/A | N/A | N/A | N/A | Can't tell | Yes |
| **4. QUANTITATIVE DESCRIPTIVE STUDIES** | 4.1. Is the sampling strategy relevant to address the research question? | N/A | N/A | Yes | Yes | N/A | N/A | N/A | Yes | Yes | Yes | Yes | Yes | Yes | N/A | N/A |
|  | 4.2. Is the sample representative of the target population? | N/A | N/A | Can't tell | Can't tell | N/A | N/A | N/A | Can't tell | Can't tell | Can't tell | Yes | Yes | Can't tell | N/A | N/A |
|  | 4.3. Are the measurements appropriate? | N/A | N/A | Yes | Yes | N/A | N/A | N/A | Yes | Yes | Yes | Yes | Yes | Yes | N/A | N/A |
|  | 4.4. Is the risk of nonresponse bias low? | N/A | N/A | No | Yes | N/A | N/A | N/A | No | No | Yes | Yes | Yes | Can't tell | N/A | N/A |
|  | 4.5. Is the statistical analysis appropriate to answer the research question? | N/A | N/A | Yes | Yes | N/A | N/A | N/A | Yes | Yes | Yes | Yes | Yes | Yes | N/A | N/A |
| **5. MIXED METHODS STUDIES** | 5.1. Is there an adequate rationale for using a mixed methods design to address the research question? | N/A | N/A |  | Yes | N/A | N/A | N/A | N/A | N/A | N/A | N/A | N/A | N/A | N/A | N/A |
|  | 5.2. Are the different components of the study effectively integrated to answer the research question? | N/A | N/A | N/A | Yes | N/A | N/A | N/A | N/A | N/A | N/A | N/A | N/A | N/A | N/A | N/A |
|  | 5.3. Are the outputs of the integration of qualitative and quantitative components adequately interpreted? | N/A | N/A | N/A | Yes | N/A | N/A | N/A | N/A | N/A | N/A | N/A | N/A | N/A | N/A | N/A |
|  | 5.4. Are divergences and inconsistencies between quantitative and qualitative results adequately addressed? | N/A | N/A | N/A | Yes | N/A | N/A | N/A | N/A | N/A | N/A | N/A | N/A | N/A | N/A | N/A |
|  | 5.5. Do the different components of the study adhere to the quality criteria of each tradition of the methods involved? | N/A | N/A | N/A | Yes | N/A | N/A | N/A | N/A | N/A | N/A | N/A | N/A | N/A | N/A | N/A |

Note: High quality studies are highlighted green, moderate quality studies are highlighted orange, and low quality studies are highlighted red.
