# Supplementary material for: Implementation of decarbonisation actions in general practice: a systematic review and narrative synthesis
Source: BMJ Open. 2025 Feb 19;15(2):e091404. doi: 10.1136/bmjopen-2024-091404 (PMC11840891; doi:10.1136/bmjopen-2024-091404)
Supplement: Supplementary file 4 [file bmjopen-2024-091404.reviewer_comments.pdf]

PEER REVIEW HISTORY

BMJ Open publishes all reviews undertaken for accepted manuscripts. Reviewers are asked to complete a checklist review form and are provided with free text boxes to elaborate on their assessment. These free text comments are reproduced below.

ARTICLE DETAILS

Title (Provisional)

Implementation of decarbonisation actions in general practice – a systematic review and narrative synthesis

Authors

Nunes, Ana Raquel; Karaba, Florence; Geddes, Olivia; Bickerton, Abi; Atherton, Helen; Dahlmann, Frederik; Eccles, Abi; Gregg, Michael; Spencer, Rachel; Twohig, Helen; Dale, Jeremy

---

VERSION 1 - REVIEW

Reviewer 1

Name Pickles, Kristen

Affiliation University of Sydney, Centre for Values, Ethics and the Law in Medicine

Date 20-Sep-2024

COI Nil

Thanks for the opportunity to review this summary of decarbonisation actions in primary care, I read it with much interest. The authors write clearly on this important topic, particularly the introduction, and fantastic to see patient and public involvement. My overall question is whether this review is better formulated as a scoping review rather than a systematic review, simply due to the non-specific nature of the research question/outcomes.

Specific comments:

METHODS

• Please structure eligibility criteria according to the PICO framework and define eligible 'decarbonisation actions' – were any types of actions/interventions excluded? Were both bottom up and top down interventions (micro, meso, macro level) considered eligible?

• Addressing the above may help to clarify the primary outcome.

• Please include more detail on the specific data extracted and whether this was double extraction, as well as whether reviewers independently graded QA and checked consensus

• I imagine that there would only be a small number of non-English studies, if any – perhaps the authors could consider checking and including these studies for completeness (using translation software to determine eligibility)

• I was surprised to see few papers on telehealth, deprescribing, respiratory inhalers, single use disposables

RESULTS

• Overall, the presentation of the Results is quite general and lacking in specific detail – for example, were there any differences observed across the various settings? (I recognise all are HIC, but likely differently funded and organised healthcare systems with different policies, net zero initiatives etc?)

• I found the 'types of actions' very limited in detail despite being the focus of the review. For example, I believe it would be helpful for the reader to understand how these actions were identified, e.g. 'revising medical prescriptions to prevent overmedication' –were these data from qual interviews? Survey? Was the action evaluated for effectiveness?

• At times it was not clear if the presented results were the author's words/interpretation or if being reported from the included articles

o E.g. 'knowledge and awareness of climate change and its health impacts among GP clinicians are crucial for promoting decarbonisation actions. Many clinicians acknowledge and feel uncomfortable discussing it with patients' (no reference).

• I was interested in more detail re specific examples of financial incentives – what they were (for individual providers? At practice level?) and how they facilitated decarbonisation actions. Same for supportive policies.

• Little sense of 'implementation' – how were the various strategies implemented in practice, what worked and didn't work, 'high costs and resource constraints' are mentioned but what exactly were these and how/were they measured?

• Suggestion to more explicitly relate the 'patient and community engagement' section back to the review question ('to summarise existing literature on the implementation of decarbonisation actions, to outline the actions being implemented, factors influencing decarbonisation).

DISCUSSION

• It would be great to include some discussion of removing low value care as a source of unnecessary emissions in primary care e.g. inappropriate testing, prescribing, etc. and associated co-benefits, as well as need to identify most impactful and cost-effective strategies for rapid decarbonisation

---

**Reviewer**	**2**
**Name**	**Gray, William**

**Affiliation**	**NHS Improvement, Getting It Right First Time**
**Date**	**25-Nov-2024**
**COI**	**None**

This is a very well written paper on an important topic. The area of research is expanding rapidly and this is a timely addition. I only have some minor comments:-

• The introduction is well written, but I think the author need more justification of why look at implementation specifically. The authors make a good case for the role of primary care, but not for why they want to look at implementation specifically. Why is implementation the key issue, rather than developing and piloting new initiatives or evaluating the success of existing initiatives? The authors hint at this by talking about primary care's disparate nature, but need to expand on the topic.

• In the introduction, the authors make a distinction between primary care and general practice. They may may want to define these terms in the methods section under eligibility criteria. They are often used by others interchangeably.

• It is good to see behavioural change frameworks being discussed in he context of implementation. However, I wonder if these could come earlier on in the discussion and even used as a framework to structure the discussion section, which tends to jump around a little.

VERSION 1 - AUTHOR RESPONSE

Responses to Reviewers

**Reviewer #1:** Thanks for the opportunity to review this summary of decarbonisation actions in primary care, I read it with much interest. The authors write clearly on this important topic, particularly the introduction, and fantastic to see patient and public involvement.	**The authors thank the reviewer for these comments and addresses specific questions below.**
RESULTS	
Overall, the presentation of the Results is quite general and lacking in specific detail – for example, were there any differences observed across the various settings? (I recognise all are HIC, but likely differently funded and organised healthcare systems with different policies, net zero initiatives etc?)	**The 'Results' section has been revised providing more specific detail and addressing the points mentioned.**

I found the 'types of actions' very limited in detail despite being the focus of the review. For example, I believe it would be helpful for the reader to understand how these actions were identified, e.g. 'revising medical prescriptions to prevent overmedication' –were these data from qual interviews? Survey? Was the action evaluated for effectiveness?	**The characteristics of included studies (i.e., country, setting and participants, and study design) are included in Table 1. The 'Type of decarbonisation actions' heading mentioned in the 'Results' section has been expanded to address the comment.**
At times it was not clear if the presented results were the author's words/interpretation or if being reported from the included articles<br>o E.g. 'knowledge and awareness of climate change and its health impacts among GP clinicians are crucial for promoting decarbonisation actions. Many clinicians acknowledge and feel uncomfortable discussing it with patients' (no reference).	**The 'Results' section text has been revised throughout to address the comment. For example: "**Knowledge and awareness of climate change and its health impacts among general practice clinicians were identified as crucial for promoting decarbonisation actions [32,35,41]. However, there was evidence that while many clinicians acknowledged the existence and threat of climate change, they may lack specific knowledge and felt uncomfortable discussing it with patients due to insufficient training and a lack of practical tools [38]."
I was interested in more detail re specific examples of financial incentives – what they were (for individual providers? At practice level?) and how they facilitated decarbonisation actions. Same for supportive policies.	**The following text has been added:** "Financial incentives at both individual and practice levels facilitated actions such as upgrading facilities and adopting sustainable practices [35,36]. However, specific examples of these incentives were often vague. Supportive policies were also essential for the adoption of decarbonisation actions, with barriers such as the lack of clear guidance in some regions hindering widespread implementation.[35,36]. In some cases, regional policies and frameworks, such as the WONCA declaration, provided guidance and motivated GPs to integrate climate change considerations into their practices [35,36]. Nonetheless, the lack of clear and region-specific directives hindered broader implementation. Effective decarbonisation also required system-level changes, including better networking and centralisation of sustainability efforts [31,35]. For a summary, see Table 2."
Little sense of 'implementation' – how were the various strategies implemented in practice, what worked and didn't work,	**The following text has been added:**<br>"However, high costs and resource constraints limited the ability of practices to adopt

‘high costs and resource constraints’ are mentioned but what exactly were these and how/were they measured?	sustainable measures; financial support and cost-effective solutions were needed to overcome these barriers [31,42]. Such barriers were frequently cited but seldom quantified, with few studies providing detailed evaluations of these costs or proposing cost-effective alternatives.”
Suggestion to more explicitly relate the ‘patient and community engagement’ section back to the review question (‘to summarise existing literature on the implementation of decarbonisation actions, to outline the actions being implemented, factors influencing decarbonisation).	**The following text has been revised:** “Specifically, this review summarises and synthesises existing literature, identifies factors influencing decarbonisation (e.g., patient and community engagement), highlights evidence gaps, and outlines questions for future research.”
**Reviewer #2:** This is a very well written paper on an important topic. The area of research is expanding rapidly and this is a timely addition. I only have some minor comments:-	**The authors thank the reviewer for these comments and addresses specific questions below.**
The introduction is well written, but I think the author need more justification of why look at implementation specifically. The authors make a good case for the role of primary care, but not for why they want to look at implementation specifically. Why is implementation the key issue, rather than developing and piloting new initiatives or evaluating the success of existing initiatives? The authors hint at this by talking about primary care’s disparate nature, but need to expand on the topic.	**The following text has been added:** “Despite these opportunities, scoping searches identified no prior systematic reviews examining the implementation of decarbonisation actions in general practice. The aim of this study is to address this gap in knowledge by systematically exploring the existing body of empirical research on the implementation of decarbonisation actions in general practice. Specifically, this review summarises and synthesises existing literature, identifies factors influencing decarbonisation, highlights evidence gaps, and outlines questions for future research. By examining how and why decarbonisation actions are implemented, this review seeks to inform the commissioning and delivery of general practice services, ultimately facilitating the transition toward sustainable healthcare.”
In the introduction, the authors make a distinction between primary care and general practice. They may may want to define these terms in the methods	**The following text has been added to the ‘Introduction’ section:** “In this review, we define primary care as comprising general practice, community pharmacies, dental services, and eyecare through optometry, with general practice described as a primary

section under eligibility criteria. They are often used by others interchangeably.	care service delivered by general practitioners (GPs) and the multi-disciplinary teams who are based within general practice.”
It is good to see behavioural change frameworks being discussed in the context of implementation. However, I wonder if these could come earlier on in the discussion and even used as a framework to structure the discussion section, which tends to jump around a little.	**The ‘Discussion’ section has been revised. Behavioural change frameworks such as the NPT and TDF, provide valuable tools. By systematically addressing the cognitive, affective, and environmental determinants of implementation, these frameworks can advance understanding of the dynamic processes driving decarbonisation in general practice. The NPT and TDF frameworks are used throughout the overall project, of which this systematic review is one component.**

VERSION 2 - REVIEW

Reviewer **1**

Name **Pickles, Kristen**

Affiliation **University of Sydney, Centre for Values, Ethics and the Law in Medicine**

Date **16-Jan-2025**

COI

Thank you for your consideration and actioning of reviewer comments, the manuscript is shaping up nicely and I believe will be an interesting read for the journal audience. No further comments from me, best wishes for the publication.

VERSION 2 - AUTHOR RESPONSE

Responses to Reviewers

**Reviewer #1:** Thanks for the opportunity to review this summary of decarbonisation actions in primary care, I read it with much interest. The authors write clearly on this important topic, particularly the introduction, and fantastic to see patient and public involvement.	**The authors thank the reviewer for these comments and addresses specific questions below.**

METHODS	
Please structure eligibility criteria according to the PICO framework and define eligible 'decarbonisation actions' – were any types of actions/interventions excluded? Were both bottom up and top down interventions (micro, meso, macro level) considered eligible?	**This has been addressed. The following text has been added:** "The eligibility criteria were structured according to the PICO framework. Population: studies investigating decarbonisation actions in general practice (or equivalent in non-UK settings). Intervention: any decarbonisation actions aimed at reducing carbon emissions within primary care. Comparator: current decarbonisation actions. Outcome: the extent and effectiveness of decarbonisation actions and factors influencing their implementation.<br><br>Eligible decarbonisation actions were defined as initiatives aimed at reducing carbon emissions within general practice settings. Bottom-up (micro-level) and top-down (meso- and macro-level) dimensions were considered eligible."
Addressing the above may help to clarify the primary outcome.	**The following text has been revised and added:** "The primary outcomes of interest were the types of decarbonisation actions implemented, including telehealth, deprescribing, respiratory inhalers, and single-use disposables. Secondary outcomes included factors influencing the adoption, implementation and integration of decarbonisation actions at institutional, organisational, professional and patient spheres."
Please include more detail on the specific data extracted and whether this was double extraction, as well as whether reviewers independently graded QA and checked consensus	**The following text has been added:** "Data extraction included study characteristics, intervention details, outcomes, and implementation factors."; "Double data extraction was performed to ensure accuracy."
I imagine that there would only be a small number of non-English studies, if any – perhaps the authors could consider checking and including these studies for completeness (using translation software to determine eligibility)	**The following text has been added:** "Non-English studies were identified and screened using translation software to determine eligibility."
I was surprised to see few papers on telehealth, deprescribing,	

respiratory inhalers, single use disposables	
RESULTS	
Overall, the presentation of the Results is quite general and lacking in specific detail – for example, were there any differences observed across the various settings? (I recognise all are HIC, but likely differently funded and organised healthcare systems with different policies, net zero initiatives etc?)	**The 'Results' section has been revised providing more specific detail and addressing the points mentioned.**
I found the 'types of actions' very limited in detail despite being the focus of the review. For example, I believe it would be helpful for the reader to understand how these actions were identified, e.g. 'revising medical prescriptions to prevent overmedication' –were these data from qual interviews? Survey? Was the action evaluated for effectiveness?	**The characteristics of included studies (i.e., country, setting and participants, and study design) are included in Table 1. The 'Type of decarbonisation actions' heading mentioned in the 'Results' section has been expanded to address the comment.**
At times it was not clear if the presented results were the author's words/interpretation or if being reported from the included articles o E.g. 'knowledge and awareness of climate change and its health impacts among GP clinicians are crucial for promoting decarbonisation actions. Many clinicians acknowledge and feel uncomfortable discussing it with patients' (no reference).	**The 'Results' section text has been revised throughout to address the comment. For example: "**Knowledge and awareness of climate change and its health impacts among general practice clinicians were identified as crucial for promoting decarbonisation actions [32,35,41]. However, there was evidence that while many clinicians acknowledged the existence and threat of climate change, they may lack specific knowledge and felt uncomfortable discussing it with patients due to insufficient training and a lack of practical tools [38]."
I was interested in more detail re specific examples of financial incentives – what they were (for individual providers? At practice level?) and how they facilitated decarbonisation actions. Same for supportive policies.	**The following text has been added:** "Financial incentives at both individual and practice levels facilitated actions such as upgrading facilities and adopting sustainable practices [35,36]. However, specific examples of these incentives were often vague. Supportive policies were also essential for the adoption of decarbonisation actions, with barriers such as the lack of clear guidance in some regions hindering widespread implementation.[35,36]. In

	some cases, regional policies and frameworks, such as the WONCA declaration, provided guidance and motivated GPs to integrate climate change considerations into their practices [35,36]. Nonetheless, the lack of clear and region-specific directives hindered broader implementation. Effective decarbonisation also required system-level changes, including better networking and centralisation of sustainability efforts [31,35]. For a summary, see Table 2."
Little sense of 'implementation' – how were the various strategies implemented in practice, what worked and didn't work, 'high costs and resource constraints' are mentioned but what exactly were these and how/were they measured?	**The following text has been added:** "However, high costs and resource constraints limited the ability of practices to adopt sustainable measures; financial support and cost-effective solutions were needed to overcome these barriers [31,42]. Such barriers were frequently cited but seldom quantified, with few studies providing detailed evaluations of these costs or proposing cost-effective alternatives."
Suggestion to more explicitly relate the 'patient and community engagement' section back to the review question ('to summarise existing literature on the implementation of decarbonisation actions, to outline the actions being implemented, factors influencing decarbonisation).	**The following text has been revised:** "Specifically, this review summarises and synthesises existing literature, identifies factors influencing decarbonisation (e.g., patient and community engagement), highlights evidence gaps, and outlines questions for future research."
DISCUSSION	
It would be great to include some discussion of removing low value care as a source of unnecessary emissions in primary care e.g. inappropriate testing, prescribing, etc. and associated co-benefits, as well as need to identify most impactful and cost-effective strategies for rapid decarbonisation	**The following text has been added:** "Finally, future research could explore the role of removing low-value care, such as inappropriate testing and prescribing, as a crucial strategy for decarbonisation. Tackling unnecessary healthcare practices not only contributes to emission reductions but also provides significant co-benefits, including improved patient safety and reduced healthcare costs."
**Reviewer #2:** This is a very well written paper on an important topic. The area of research is expanding rapidly and this is a timely	**The authors thank the reviewer for these comments and addresses specific questions below.**

addition. I only have some minor comments:-	
The introduction is well written, but I think the author need more justification of why look at implementation specifically. The authors make a good case for the role of primary care, but not for why they want to look at implementation specifically. Why is implementation the key issue, rather than developing and piloting new initiatives or evaluating the success of existing initiatives? The authors hint at this by talking about primary care's disparate nature, but need to expand on the topic.	**The following text has been added:** "Despite these opportunities, scoping searches identified no prior systematic reviews examining the implementation of decarbonisation actions in general practice. The aim of this study is to address this gap in knowledge by systematically exploring the existing body of empirical research on the implementation of decarbonisation actions in general practice. Specifically, this review summarises and synthesises existing literature, identifies factors influencing decarbonisation, highlights evidence gaps, and outlines questions for future research. By examining how and why decarbonisation actions are implemented, this review seeks to inform the commissioning and delivery of general practice services, ultimately facilitating the transition toward sustainable healthcare."
In the introduction, the authors make a distinction between primary care and general practice. They may may want to define these terms in the methods section under eligibility criteria. They are often used by others interchangeably.	**The following text has been added to the 'Introduction' section:** "In this review, we define primary care as comprising general practice, community pharmacies, dental services, and eyecare through optometry, with general practice described as a primary care service delivered by general practitioners (GPs) and the multi-disciplinary teams who are based within general practice."
It is good to see behavioural change frameworks being discussed in the context of implementation. However, I wonder if these could come earlier on in the discussion and even used as a framework to structure the discussion section, which tends to jump around a little.	**The 'Discussion' section has been revised. Behavioural change frameworks such as the NPT and TDF, provide valuable tools. By systematically addressing the cognitive, affective, and environmental determinants of implementation, these frameworks can advance understanding of the dynamic processes driving decarbonisation in general practice. The NPT and TDF frameworks are used throughout the overall project, of which this systematic review is one component.**
